# Recent Advances in Life History Transition with Nematode-Trapping Fungus *Arthrobotrys oligospora* and Its Application in Sustainable Agriculture

**DOI:** 10.3390/pathogens12030367

**Published:** 2023-02-22

**Authors:** Da Wang, Nan Ma, Wanqin Rao, Ying Zhang

**Affiliations:** 1 State Key Laboratory for Conservation and Utilization of Bio-Resources in Yunnan, Yunnan University, Kunming 650032, China; 2 School of Life Science, Yunnan University, Kunming 650032, China

**Keywords:** parasitic nematodes, biological control, nematode-trapping fungi, application prospects

## Abstract

Parasitic nematodes cause great annual loss in the agricultural industry globally. *Arthrobotrys oligospora* is the most prevalent and common nematode-trapping fungus (NTF) in the environment and the candidate for the control of plant- and animal-parasitic nematodes. *A. oligospora* is also the first recognized and intensively studied NTF species. This review highlights the recent research advances of *A. oligospora* as a model to study the biological signals of the switch from saprophytism to predation and their sophisticated mechanisms for interacting with their invertebrate hosts, which is of vital importance for improving the engineering of this species as an effective biocontrol fungus. The application of *A. oligospora* in industry and agriculture, especially as biological control agents for sustainable purposes, was summarized, and we discussed the increasing role of *A. oligospora* in studying its sexual morph and genetic transformation in complementing biological control research.

## 1. Introduction

Nematodes are the most abundant animals in the global soil ecosystem; they constitute a large proportion of the soil community and participate in a wide range of ecological interactions, including nitrogen mineralization by free-living nematodes and parasitism with animals, plants, and fungi [1]. Among them, plant-parasitic nematodes cause an estimated annual loss ranging from $80 billion to $157 billion around the world [2,3], as well as have a significant impact on the agricultural industry [4].

Chemical treatment, immunological modulation, and biological control are the major approaches used to control these parasitic nematode infections [5], of which chemical anthelmintics are commonly used in agriculture for deworming [6]. However, chemical treatments cause subsequent drug resistance and serious environmental pollution [7], including issues such as soil compaction, water pollution, and chemical residues in the environment. Moreover, chemical residues may affect human health through the environment and by food contamination [6,8,9]; therefore, farmers must acquire relatively inexpensive and environmentally friendly nematode management techniques at the earliest point possible to deal with these annoying plant-parasitic nematodes [10]. Management of resistance and sustainability are important considerations in the development of novel nematode control strategies [5].

Nematode-trapping fungi (NTF) are a group of specialized microbial predators that can recognize the presence of nematodes and have the capacity to trap and digest nematodes [11,12]. By producing extensive hyphal traps, such as constricting rings, adhesive knobs, and networks, they can trap and hold living nematodes and then utilize their nutrients for growth [13]. Both nematodes and NTFs have a wide range of species diversity in the soil ecosystem, and the majority of them exhibit sympatric distribution and close relationships with each other [12]. This peculiar feature of killing living nematodes by their natural life strategy makes NTFs the ideal alternative for the chemical control of parasitic nematodes [14,15], especially in the rhizosphere of plants, and, hence, their interactions exert a significant effect on agriculture and forestry [16,17].

One of the most prevalent and common NTFs in the environment is *Arthrobotrys oligospora* Fres. 1852, which is also the first recognized and intensively studied NTF species [18]. Its efficient trapping ability has rendered *A. oligospora* an ideal candidate for the control of plant- and animal-parasitic nematodes. For instance, the ability of *A. oligospora* C-2197 to manage nematode populations provides a feasible concept for the management of tomato production in a greenhouse environment [14]. Moreover, *A. oligospora* (MRDS 300) is a promising biocontrol agent for *Meloidogyne incognita* [19].

The wide ecological distribution and indications of historical differentiation, recent hybridization, and a significant genetic/phenotypic variation of natural populations in *A. oligospora* have emphasized the importance of evaluating the specificity of NTF–PPN interactions [20]. Moreover, future biocontrol applications call for the growth and reproductive abilities of the selected strains [20,21,22] because the potential recombination between divergent populations has indicated the possibility of improving both the survival of the introduced genotype with higher nematode pathogenesis and the biocontrol applications of native strains by generating recombinant genotypes [22]. In addition, the various abilities of trap formation by *A. oligospora* natural strains subsequently correlated with high performance in prey killing, demonstrating that the interaction of *A. oligospora* as a model organism with nematodes is a sign of natural adaptation in generalist predators of the NTFs [12].

An in-depth understanding of the interaction between *A. oligospora* and nematodes could aid in the control of nematode populations and the development of new biological control products [23]. With the first sequenced genome of the NTF *A. oligospora* (strain ATCC24927), omics studies of nematophagous fungi have provided novel insights into the biological signals of the switch from saprophytism to predation and their sophisticated mechanisms for interacting with their invertebrate hosts [24,25,26,27,28]. In this review, we summarize the recent advances in understanding the interaction of *A. oligospora* as a model organism with nematodes. In particular, we describe their entire interaction from initial attraction, recognition and identification, trap formation, adhesion, penetration, and digestion and summarize the recent applications of this fungus in biocontrol and industry.

## 2. The Process of Attracting Nematodes by *A. oligospora*

Because nematodes can move quickly, but NTFs cannot, NTFs must implement some steps to attract nematodes [29], which will result in subsequent invasion and digestion. Through this approach, NTFs can lure nematodes to stay close by releasing volatile metabolites [19,30], allowing the NTFs to launch the transition from a saprophytic to a parasitic existence and thus complete the nematode predation process [11].

Under natural conditions, *A. oligospora* and *Caenorhabditis elegans* have been observed to be encountering and interacting frequently [12]. Moreover, the attraction of *A. oligospora* is neither conserved nor unique to *C. elegans*; in fact, the compounds released by *A. oligospora* or other NTFs are still attractive to other worms [30]. Meanwhile, the metabolites secreted by *A. oligospora*, such as methyl 3-methyl-2-butenoate (MMB), (±)2-methyl-1-butanol (MB), 2,4-dithiapentane (DTP), S-methyl thioacetate, dimethyl disulfide, and some other natural compounds containing furan rings, are volatile [11,30] and mediate the interaction between *A. oligospora* and nematodes in the long-term evolution [19]. It was discovered that three metabolites with a furan ring and similar molecular weights attracted *C. elegans* during the preliminary chemotaxis bioassay, which included 2(5H)-furanone, furan-2-yl methanol, and furan-2-carbaldehyde [11]. This finding indicates that NTFs can chemically lure their prey into the trap, and the volatile signals are important in the capture process [11]. Furthermore, some metabolites even mimic the worm’s food and sexual cues to attract it to the side of NTFs [30]. The larvae and males of *C. elegans* do not exhibit strong attraction to this smell, which is related to the gender and developmental stage-specific attraction. Similarly, adult females or hermaphroditic individuals of other *C. elegans* are also strongly attracted to MMB, whereas males demonstrate rejection. Experiments have also shown that MMB interferes with *Caenorhabditis* spp. mating [30].

Nematodes have associated olfactory nerves, such as AWC neurons, and corresponding odor-recognition genes for detecting the volatile chemicals of *A. oligospora* [30], and AWC neurons respond to only lower odorant concentrations [31]. Experiments on attraction to scents that communicate similar food or sexual signals and genetic screening of AWC neurons and single-cell transcription have demonstrated that two AWC olfactory neurons in *C. elegans* mediate the attraction of NTFs to the nematode [30]. Olfactory cues are used by nematodes for a variety of purposes, including finding mates [32], avoiding danger [33], and locating food sources [34,35,36]. *C. elegans* has long been known to be attracted by several volatile compounds, including alcohols, chemical substances with sulfur bonds [30], metabolites with furan rings [11], and tiny chemical molecules such as triazoles [37,38]. Studies have also demonstrated that almost all the metabolites produced by NTFs to attract nematodes are tiny organic molecules and liposoluble [11,30], because these fat-soluble substances more easily pass through the cell membrane of nematodes and trigger nematodes to exhibit an odor stimulation reaction [39,40].

In some animals, the same odorant can elicit pleasant or repellent responses depending on its concentration [31]. Similarly, changes in concentration can exert an impact on the nematode’s perception of volatile compounds, ranging from attraction to avoidance behavior. For instance, *C. elegans* is attracted to low concentrations of isoamyl alcohol [38], but an increase in the concentration causes repulsion and avoidance responses [31]. Moreover, 2,4,5-trimethylthiazole [31], benzaldehyde [31,41], and other compounds are similar to isoamyl alcohol [42], and furan-2-ylmethanol is appealing to feeding nematode fungus when undiluted, but it is repellent to nematodes at low quantities [11]. Table 1 lists some small chemical molecules that can attract or repel nematodes.

Numerous studies have investigated the mechanisms underlying the interactions between compounds and nematodes; however, studies focusing on *A. oligospora* can more comprehensively and precisely shed light on the interactions between the majority of compounds and nematodes.

## 3. Strategies of *A. oligospora* for Identifying Nematodes

The recognition of nematodes by *A. oligospora* can be approached from two aspects. On the one hand, ascarosides secreted by nematodes can not only attract *A. oligospora* but also help *A. oligospora* recognize nematodes [43]. Yen-Ping Hsueh et al. investigated whether ascarosides induced the formation of traps for NTFs and discovered that some of these small molecules exhibited strong trapping activity in several nematode-trapping fungi, representing an example of predator–prey coevolution and eavesdropping on prey communication [43]. On the other hand, *A. oligospora* can produce a lectin, which was initially identified in the traps of *A. oligospora* [44], and the initial examples of a lectin-mediated interaction in fungi–host interactions were from research using *A. oligospora*. Several or all sugar residues found in the nematode epidermis can be recognized by lectins [23,45]. Moreover, the MAPK signaling pathway of *A. oligospora* is related to its recognition of nematodes at the molecular level. It has been demonstrated that nematode signaling activates the Fus3 MAPK pathway in *A. oligospora* [46]. At the genetic level, Liang et al. discovered that by knocking out the *AoMad1* gene in the cell wall of *A. oligospora*, nitrogen source substances such as nitrate could more easily stimulate fungi to produce a three-dimensional bacterial net [47]. Consequently, it was hypothesized that the presence of *aomead1* would also aid fungi to correctly identify nematodes, to promote the next step [47].

## 4. Mechanisms of Trap Formation in *A. oligospora*

Here, we summarize the current knowledge of the regulatory mechanism of trap formation in *A. oligospora*, including the triggers of trap development and trap morphogenesis.

### 4.1. Triggers of Trap Development in NTF

The term “predation” is used to describe the idea that an individual of one species (predator) kills and consumes the biomass of an individual of another species (prey) [48]. Predation can impose selective pressure on both the predator and prey. In addition to carnivorous animals, there are carnivorous plants, carnivorous fungi, and carnivorous bacteria. In a nutrient-deficient environment, carnivorous plants such as pitcher grass and the Venus flytrap have evolved predatory organs and supplement their nutrient requirement by attracting, capturing, and killing a prey, followed by digestion and absorption [23]. Most fungi growing in nitrogen-deficient environments have also developed predatory organs to capture nematodes for nitrogen requirement [30]. Some NTFs form traps spontaneously, but, still, most of them need abiotic and biotic stimuli. Factors such as the carbon dioxide content in the environment, carbon source, nitrogen source, light, and phosphate level will inhibit the production of traps. In addition to these abiotic factors, different biotic factors will also influence the formation of traps [29].

*A. oligospora* was characterized by Fresenius in 1852. It was initially considered to be a saprobe that obtained nutrition from rotten organic matrix for survival. When the NTF felt the nearby nematode, it began transition from a saprophytic to parasitic life to infect the nematode [23]. In 1973, Nordbring-Hertz confirmed that small peptides or amino acids could trigger trap formation when NTFs were cultured in a low-nutrient medium [49]. Then, it was found that there are more than 100 types of scaris lumbricoides secreted by nematodes, and some of them with ascarosides containing 7- and 9-carbon side chains can induce *A. oligospora* in a low-nutrient environment to produce traps [50]. In addition to nematodes, certain bacteria can induce trap morphogenesis [51,52]. Ammonia metabolites in volatile organic compounds secreted by bacteria can also induce *A. oligospora* to produce traps, and a variety of nematodes also produce traps by ammonia [53]. Ammonia is a urea metabolite and functions as a signaling molecule to accelerate the switch from a saprophytic to a predatory lifestyle in NTF [54]. Certain bacteria can mobilize NTF to reduce predation pressure by producing and releasing urea [55]. In 2016, a study reported that bacterial biofilm formation on hyphae had a relationship with trap formation in cured *Arthrobotrys* [56].

### 4.2. Trap Morphogenesis

The sign of the transformation from a saprophytic to a parasitic life state indicates the production of predatory organs. The nematode-trapping fungus *A. oligospora* captures nematodes using three-dimensional adhesive network traps [57]. These traps are developed into numerous anastomoses of hyphal loops by the formation and fusion of newly developed loops, and each initial loop develops perpendicularly from the parent hypha, which finally becomes a three-dimensional network [58]. The involvement of dense bodies and the unique ability to capture nematodes differ these cells from typical hyphal cells [59]. The synthesis of these dense bodies begins at a very early stage of trap formation. Interestingly, in *A. oligospora*, the first cell of the newly formed trap has dense bodies already, which displays all the properties of a mature trap [60,61]. These dense bodies are cytosolic organelles containing catalase and d-amino acid oxidase and thus are peroxisomal in nature. They may function as energy-supplying or structural components to the invading hyphae, and they might be involved in the adhesion of nematodes and the development of trophic hypha after the penetration of the nematode cuticle [61].

In addition to these dense bodies, another distinguishing characteristic of trap cells is that they are coated with a fibrillar adhesive, while the hyphal cells are not. The electron-dense fibrils containing neutral sugars, uronic acids, and proteins construct the adhesive layer [62]. During the first stage of contact with nematodes, immobilization of the prey, which facilitates the consequent digestion, was accomplished by the adhesive layer. In addition, several other different mycelial structures, such as conidial traps, hyphal coils, and the recently discovered appressoria could be generated by *A. oligospora* [60]. In addition to mature traps, nematodes can be captured on the first formed parent branches [62]. The trap structures could also form from the surface of conidia directly upon germination without an intermediate hyphal phase, which are called conidial traps. They are found, up to now, to be developed only with the presence of natural substrates such as cow faeces, soil, or soil extracts, and not in pure culture, suggesting that environments with strong competition for nutrients are favorable for these structures [63].

### 4.3. Regulatory Mechanism of Trap Formation in A. oligospora

Genome, proteome, transcriptome, and other related omics studies have significantly broadened our understanding of the molecular background of nematode-eating fungi and laid a good foundation for understanding and investigating the molecular mechanism underlying the peculiar transition from the saprophytic to parasitic life mode, trap formation, and disease [62]. *A. oligospora* was sequenced in 2011, which revealed that it contains a 40.07-Mb assembled sequence with 11,479 predicted genes [64]. A comparative analysis demonstrated that *A. oligospora* shared several more genes with pathogenic fungi than with nonpathogenic fungi. Through comparative proteome analysis and RT-PCR verification, it was found that trap formation involved translation, amino acid metabolism, carbohydrate metabolism, and cell wall and membrane biogenesis [62]. This suggests that multiple biological processes are involved in the trap formation, which is also highly energy-consuming [30]. Recent research has confirmed that the G-protein signaling pathway regulates the formation of traps in *A. oligospora* [65]. A total of 20 genes involved in trap formation were found to be related to the G-protein signaling pathway. Of these, seven *Rgs* genes (*FlbA*, *RgsA*, *RgsB*, *RgsB2-1*, *RgsB2-2*, *RgsB2-3*, and *RgsC*) negatively regulate G-protein signal transduction, and it was observed that intracellular cAMP levels, which affect mycelial growth, stress resistance, conidiation, trap formation, and nematocidal activity, were also negatively regulated by *Rgs* genes [66]. In particular, the Δ*AoFlbA* mutant could not produce conidia and traps. Meanwhile, *AoFlbA* was found to regulate amino acid metabolism and affect trap formation in *A. oligospora* by transcriptome analysis, during which amino acid metabolic and biosynthetic processes were significantly enriched [66]. Furthermore, there are nine genes (*Gas1*, *Ras2*, *Ras3*, *Rheb*, *Rab-7A*, *Rab-2*, *Rho2*, *Rac*, and *Cdc42*) related to conserved groups of the superfamily of small GTPases that comprise signal transducers regulating multiple cellular functions [26,27,67]. Recently, knockout and the transcription of genes *AoRab-7A* and *AoRab-2* identified the roles of two Rab GTPases in *A. oligospora*. When *AoRab-7A* was disrupted, *A. oligospora* almost lost its ability to produce conidia, and four sporulation-related genes (*AbaA*, *FluG*, *Hyp1*, and *VosA*) were negatively regulated. Moreover, the Δ*AoRab-7A* mutants could not produce traps or capture nematodes. However, the disruption of *AoRab-2* exerted only a slight impact on conidiation, and trap formation remained unaffected [26,27,67]. In another study, three orthologous Ras GTPases (*Ras2*, *Ras3*, and *Rheb*) were identified in *A. oligospora*, and the disruption of all three genes affected the growth, sporulation, adaptation, and predatory ability of *A. oligospora* [27]. Deletion of *Aoras2* and *Aorheb* also further played vital roles in biological process, such as the mitochondrial activity and the biosynthesis of secondary metabolites [27]. Recent research also demonstrated that three RHO GTPases (RHO2, RAC, and CDC42) affected mycelial growth, lipid accumulation, DNA damage, conidiation, trap formation, pathogenicity, and stress resistance in *A. oligospora* [26]. In addition, genes related to the G-protein signaling pathway involved in trap formation include *gpb1* (G-protein β subunit) [30], *gas1* (GAS protein) [65], *glo3* (ARF GTPase activator) [66], and *ric8* (resistance to inhibitors of cholinesterase) [67]. Interestingly, all 20 gene mutants exhibited reduced trap formation ability [24]. In all, G-protein signaling plays essential roles in vegetative growth, development, and pathogenicity of *A. oligospora*, and its importance in the lifestyle switch of NTF should be further studied.

In addition to the G-protein signaling pathway, trap formation is also controlled by the mitogen-activated protein kinase (MAPK) signaling pathway [68]. There are three major MAPK cascades in yeast and ascomycetes, including cell wall integrity, pheromone response and filamentous growth, and hyperosmolarity pathways [62]. The cell wall integrity pathway, including three MAPKs, viz., Bck1, Mkk1/2, and Slt2, is a primary signaling pathway for fungal pathogenesis. Bck1 and Mkk1 are upstream of Slt2, and they positively regulated sporulation and the ability to produce mycelial traps for nematode predation [68,69]. A recent study showed that disruption of *AoSlt2* and *MhSlt2* resulted in reduced mycelial growth and an inability to produce conidia and nematode-trapping structures in *A. oligospora* and *M. haptotylum* [68]. The hyperosmolarity pathway has a typical two-domain response regulator protein—Ssk1 in the two-component signal transduction system. The Δ*Aossk1* reduced 95% of conidial production and increased a remarkable trap formation and predation efficiency in *A. oligospora* [70]. Hog1 and Msb2 are downstream of Ssk1, and they also affected pathogenicity of *A. oligospora* [71]. Another study identified MAPK FUS3, its upstream kinase (MAPKK) STE7, and the transcription factor STE12 in *A. oligospora*. They were found to be essential for growth, but only the kinases Ste7 and Fus3 positively regulated conidiation [50]. Furthermore, Ime is a protein kinase required for various cellular processes, such as sexual reproduction. The deletion of *Aoime2* resulted in defective growth and a considerably higher number of cell separation and lower number of cell nuclei in mycelia. Meanwhile, the mutants could not produce sufficient traps and conidiation [72].

Several signaling pathway genes related to cAMP-dependent protein kinase A (cAMP/PKA) have also been found in *A. oligospora* [73]. The disruption of *ras2*, *rheb*, *ric8*, and *StuA* significantly reduced cAMP levels in the WT strain [74]. Ca^2+^/calmodulin-dependent protein kinases (CaMKs) and the low-affinity calcium uptake system were also important in trap formation [73,74,75,76]. Moreover, a conserved cellular recycling and trafficking pathway in eukaryotes called autophagy is also found to affect cell development and pathogenicity in *A. oligospora*. *Latg1* and *latg13*, *atg1*, *atg4*, and *atg5* are related to autophagy in *A. oligospora*. In addition, mutations in *latg13*, *atg1*, *atg4*, and *atg5* led to the loss of ability for trap formation in *A. oligospora* [77,78,79,80]. Besides autophagy, some cellular processes affect the formation of traps in *A. oligospora*, such as woronin body synthesis [81], RNA interference [82], glycerol biosynthesis [83], production of reactive oxygen species [84], F-box protein synthesis [85], nitrate assimilation pathway [86], pH-sensing receptor protein synthesis [87], velvet family protein synthesis [88], scaffold protein synthesis [89], lectin synthesis [90], actin synthesis [91], and malate synthase [92]. Finally, research confirms that *A. oligospora* possesses pathways related to the biosynthesis of SECs of the gene cluster AOL_s00215g, containing 11 genes. The disruption of 7 of these 11 genes (283, 281, 282, 277, 278, 279, and 280) could remarkably enhance the capacity to develop trapping devices in *A. oligospora* [93].

In addition, the morphology of trap formation in *A. oligospora* could be enhanced by producing the volatile pyrone metabolite maltol. The *AOL_s00079g496* gene in *A. oligospora* greatly inhibited the amounts of the attractant furanone (2-fold) and the attractive activity of the fungus [11,93].

## 5. Adhesion, Penetration, and Digestion

The trapping process of the nematode-trapping fungus *A. oligospora* comprises several stages, i.e., attraction, recognition, trap formation, adhesion, penetration, and digestion [94]. We primarily discuss the following steps: adhesion, penetration, and digestion.

### 5.1. Trapping and Adhesion of Nematodes by A. oligospora

The morphological structure of *A. oligospora* changes after recognizing nematodes, allowing it to produce a unique predator organ—the adhesive network. The process of adhesion begins when living and motile nematodes contact the fungal trap and are required before infection [23]. Some extracellular polymers of *A. oligospora*, primarily composed of protein and carbohydrates, are essential for the adhesion and capture of nematodes [95]. Before the adhesion layer contacts the nematodes, the polymer fibers that constitute the adhesion layer are loosely arranged. The fungus positively secretes external polymers when nematodes contact the extracellular adhesive layer, and the nematode surface is aligned by dense fibrils. The extracellular polymers recombine to connect the epidermis of the nematode with the cell wall of the fungal trap, which is beneficial for the fixation of *A. oligospora* to nematodes [96]. Another vital function of the adherent substance is that it acts as a matrix, where several extracellular toxic proteins against nematodes may be hidden [97]. Because hyphal infiltration occurs shortly after the capture of nematodes, this may improve the accuracy of the function of these lethal factors. In addition, research has shown that the aforementioned *AoMad1* is involved in synthesizing the surface adhesion proteins of insect traps. The deletion of this gene causes the mutant to lack cell surface adhesive materials, which reduces nematicidal activity [97].

### 5.2. A. oligospora Invades the Nematode Cell Wall through Protease

A solid proteic exoskeleton cuticle of nematodes protects them from environmental stresses and mechanical injury by the predators [98]. Therefore, fungi that feed on nematodes must overcome this barrier to feed on their prey. Enzymatic degradation and mechanical pressure, both of which play a dual role in the penetration of this barrier, are involved [99,100]. In the process of infecting nematodes, NTFs can produce a variety of extracellular enzymes such as collagenase, serine protease, and chitinase [100]. These enzymes can damage the epidermis of nematodes, degrade epidermal proteins, and promote infiltration and colonization [99,100,101]. To date, *A. oligospora* is found to be the only NTF that has two extracellular serum proteases during infection, viz., Aoz1 and PII [23]. PII is a serine protease that can fix free-moving nematodes and hydrolyze their epidermal proteins [101]. A previous study showed that the major virulence protease during nematode invasion is P186, and not PII [100]. Moreover, collagenase is believed to be the critical factor for NTF-infected nematodes [100]. The formation of a penetration tube mediated the insertion into the nematode cuticle by *A. oligospora*, which occurs in tandem with the secretion of enzymes [99]. The piercing tube grows into the stratum corneum of nematodes, and, under its mechanical force, the stratum corneum is first indented and finally pierced [29], and, during the penetration process, the number of dense bodies significantly decreases [102].

### 5.3. Digestion of Nematodes by A. oligospora

At the final stage of the penetration process, the attacking fungus forms an infection bulb in the penetration tube [103]. This bulb produces new trophic hyphae that colonize and digest the nematode [104]. The dense bodies gradually degrade as the infected ball and vegetative mycelium mature, but normal fungal organelles are activated, and the endoplasmic reticulum is remarkably developed [61]. The ultrastructure of the vegetative mycelium and infected ball cells is transformed into normal vegetative mycelium cells. The trophic hyphae are involved in the digestion of the captured nematodes. It is worth noting that lipid droplets increase in the vegetative hyphae later in the process, which may involve the assimilation and storage of nutrients obtained from infected nematodes [103]. Lectins are also abundant in the vegetative hyphae of infected nematodes, which are capable of storing nitrogen that can then be transported into the hyphae and used to support fungal growth [105].

## 6. The Interaction among *A. oligospora*, Nematodes, and Plants

*A. oligospora* is versatile, it has the ability to be saprophyte, nematode pathogen, and plant root colonizer [106]. Plant rhizospheres increase the density of NTF in plant roots, releasing a wide range of chemicals that affect the interactions between plants and other organisms [107,108,109]. Plants metabolize nematode pheromones and produce chemical signals that can repel nematodes and reduce the risk of nematode infection, which is a form of interaction between plants and nematodes [110]. Importantly, plants can also interact with nematodes through NTFs. For instance, the phytohormone abscisic acid affects how successfully NTFs capture nematodes, showing that communication between plants and NTFs may be possible [106]. Maltol, a substance found in large quantities in several beans and other plant sources, was recently discovered to regulate NTF to form 3D traps [11], again suggesting that interactions between plants and NTFs occurs under specific natural circumstances.

**Figure 1:**
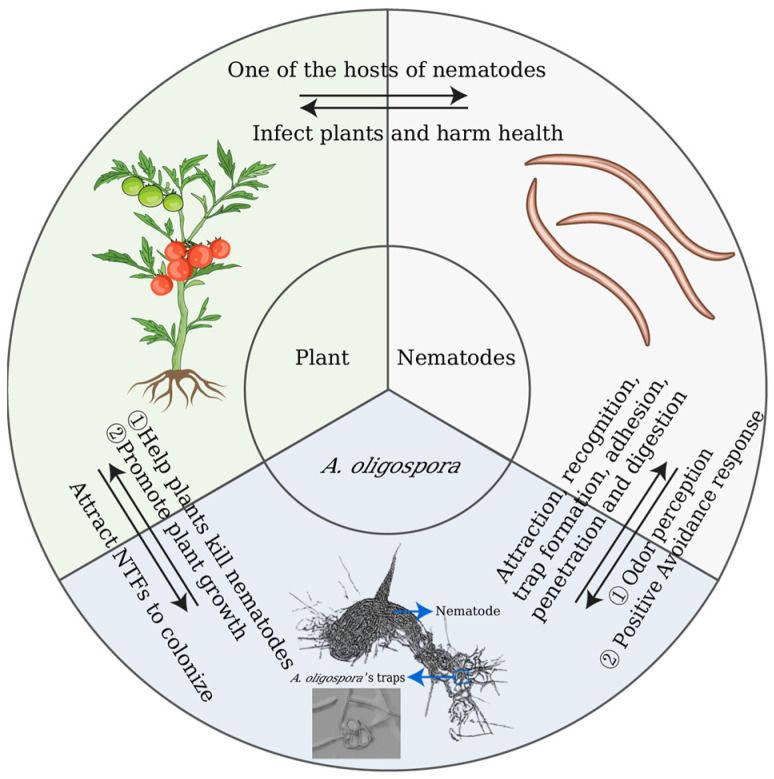
Interactions among *A. oligospora*, nematodes, and plants.

On the one hand, plant rhizospheres secrete chemicals to attract *A. oligospora* to colonize, and, correspondingly, *A. oligospora* can rapidly colonize root cells and reach the cortex, becoming a colonizer of plant roots [108]. On the other hand, correlation analysis has revealed that *A. oligospora* populations were weakly positively related to worm numbers in some situations [111]. *A. oligospora* secretes metabolites to attract predatory nematodes, reducing nematode damage to plants and forming an ecological niche among the three. Hence, the densities of NTFs in rhizospheres were slightly greater than those in root-free soil [106], and the response of root cells to NTF colonization may have significant implications for the performance of these organisms as plant-parasitic nematode biocontrol agents [112]. The presence of *A. oligospora* not only decreases the number of root-knot nematodes by the preying process but also promotes plant growth [19], such as *A. oligospora* C-2197 that exhibits root development and leaf area growth-promoting activities, as well as growth-promoting activity, on tomato plants [14]. These findings offer vivid examples of diverse predator–prey interactions in nature, playing a critical role in maintaining population composition and dynamics of both counterparts. Figure 1 depicts the interaction of *A. oligospora* and nematodes and plants, using tomatoes as an example.

## 7. Application of *A. oligospora* in Industry and Agriculture

Nematodes are abundant and live as parasites or free-living forms surviving in a variety of environments [113,114]. Parasitic nematodes have been traditionally divided into two major groups based on their hosts, i.e., plants and animals [23]. Phytoparasitic nematodes and gastrointestinal nematodes are economically the most important pathogens of agricultural products that are responsible for global agricultural losses amounting to tens of billions of dollars worldwide annually [115]. Currently, the parasitic nematode infection is still controlled by application of chemical pesticides. However, the negative impact on the environment and human health is evident [116]. Due to the harmful effects of those nematicides, there is increasing attention on other methods such as biological control [116].

NTFs are one of the biological control agents. They are widely distributed throughout the world and exist in various ecological environments, including farmland soil, garden soil, and forest soil. *A. oligospora* is one of the most important biocontrol fungi that can capture and kill a variety of nematodes [12,117]. Ecological surveys conducted to date have suggested that *A. oligospora* is the most widely distributed and most frequently isolated NTF in the environment [12]. It is extremely adaptable, populating most continents and numerous environments, such as soil, animal feces, surface waters, and heavily polluted substrates [118]. These characteristics indicate that *A. oligospora* has significant potential to be used in nematode control. At present, considering the limitation of the independent use of a single NTF in controlling phytoparasitic nematodes, combined applications that integrate multiple fungi, or fungi and chemical control combination, have attracted more attention [116]. The root-knot nematode (*M. javanica*) control technique by using both *A. oligospora* and salicylic acid that induced plant resistance activates plant defense mechanisms, which are more useful than salicylic acid or *A. oligospora* alone [119]. Furthermore, the combined use of *A. oligospora* and bacteria to eliminate nematodes has a good market prospect [120]. Studies conducted to date have demonstrated the potential of *A. oligospora* in a variety of plants, such as coffee, tomato, black pepper, cucumber, and sugar beet [121,122,123,124]. Obviously, in addition to directly killing nematodes using live fungi, nematicidal natural products from NTF or other fungi can be used as an alternative for biological control [125].

To improve the application prospect of *A. oligospora*, researchers have begun constructing genetic and genomic tools to explore nematode-trapping and -killing mechanisms, and all the above-described and identified signals and molecular mechanisms, including attraction, recognition, trap formation, adhesion, penetration, and digestion, are promising targets to be applied. To date, to improve the pathogenicity of these fungi by genetic engineering, improving their virulence factors is promising [123]. Obviously, appropriate pH levels, temperature, light intensity, and carbon and nitrogen source are crucial to the growth of *A. oligospora* [124]. Furthermore, mutagenic treatments such as low-energy ion beam implantation could generate mutants with high efficiency in trapping nematodes of *A. oligospora*, indicating a new modifying strategy to enhance virulence of fungi [126].

In addition to its use in biological control, *A. oligospora* has great prospects in industrial applications. In agriculture, besides controlling nematodes, *A. oligospora* can produce a type of phytase that is highly differentially expressed only in its parasitic stage during the development of the network. The fungal phytase has a strong capacity to enhance the release of inorganic phosphorus and soluble minerals in different feeds, indicating its potential use in feed processing in the future [127]. Moreover, scientists have investigated the in vitro degradation of asphalt by microorganisms isolated around the asphalt deposition layer and found that *A. oligospora* resulted in higher weight loss (42.83%). This finding established that *A. oligospora* is useful in the remediation of bitumen-polluted environments when the exploitation of the oil resource commences [128]. Wang et al. reported that *A. oligospora* might produce nanoparticle (NP), which has potential therapeutic applicability as an immunomodulator [129].

However, *A. oligospora* has been found as a generalist predator in the soil because it does not specifically recognize and prey on particular species of nematodes [12]. Therefore, the non-target mortality in the populations of free-living and beneficial nematodes would negatively impact the success of either agriculture or industry applications. Up to now, although little is known about the effects of fungus on non-target nematodes, while free-living nematodes were found to be relatively more sensitive to chemical pesticides compared with plant-parasitic nematodes in a study that evaluated the non-target effects of entomopathogenic nematodes [130], the quantitative experimentation and basic research on the modes of fungal–nematode interactions, host specificity, and epidemiology of target parasite nematodes are necessary. A previous study found that the massive addition of chlamydospores of nematode-trapping fungus *Duddingtonia flagrans* in feed supplements for the integrated control of gastrointestinal nematodes in sheep does not affect faecal colonization by other fungi and soil nematodes and, once deployed on pasture, does not survive for long periods in the environment [131]. Thus, it is of vital importance to monitor the target nematode populations, as the best time to apply fungi is before pest populations reach their peak, so early application can increase their effectiveness.

## 8. Concluding Remarks

*A. oligospora* is the most widely distributed and the most extensively investigated NTF in the environment. It can form three-dimensional network traps that capture nematodes. Its trapping process normally has six stages, namely attraction, recognition, trap formation, adhesion, penetration, and digestion. The development of genome, proteome, transcriptome, and other related omics has significantly broadened our understanding of trap formation, pathogenesis, and the lifestyle transition from saprophyte to parasite of this special type of fungus. This knowledge is extremely important for improving the engineering of this species as an effective biocontrol fungus. Studies conducted to date have demonstrated that *A. oligospora* has high efficiency in potential application to control both plant- and animal-parasitic nematodes. In addition to its use as a biocontrol agent, *A. oligospora* has new application prospects in the industry, such as producing natural nanoparticles (NPs), remediation of bitumen-contaminated environments, and feed production.

The current research primarily focuses on *A. oligospora* under its asexual morph, as it is assumed that *A. oligospora* has the ability to recombine and generate mutations under environmental stress, the selection of WT strains from stressed soils as biocontrol candidates, or strains with recombinant genotypes by crossing with native strains and strains with powerful parasitizing ability are the next promising direction for application. Therefore, laboratory mating of *A. oligospora* is essential to provide an important basis for the genetic transformation of crucial virulence genes and understand the ecological adaption of this fungus.

## Figures and Tables

**Table 1:** Small chemical molecules that attract or repel nematodes and their properties.

Chemical Structural Formula	Name	Molecular Formula	Attract or Repel	Solubility	Ref.
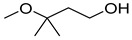	Methyl 3-Methyl-2-Butenoate	C_6_H_14_O_2_	Attract	Liposoluble	[29]
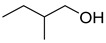	(±)2-Methyl-1-Butanol	C_5_H_12_O	Attract	Liposoluble	[29]
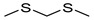	2,4-Dithiapentane	C_3_H_8_S_2_	Attract	Liposoluble	[29]
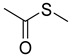	S-Methyl Thioacetate	C_3_H_6_OS	Attract	Liposoluble	[29]
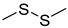	Dimethyl Disulfide	C_2_H_6_S_2_	Attract	Liposoluble	[29]
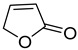	2(5H)-Furanone	C_4_H_4_O_2_	Attract	Liposoluble	[11]
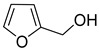	Furan-2-Ylmethanol	C_5_H_6_O_2_	Attract	Liposoluble	[11]
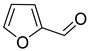	Furan-2-Carbaldehyde	C_5_H_4_O_2_	Attract	Liposoluble	[11]
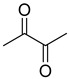	Diacetyl (low)	C_4_H_6_O_2_	Attract	Liposoluble	[38]
	Diacetyl (high)		Repel		[31,42]
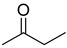	2-Butanone	C_4_H_8_O	Attract	Hydrosoluble/Liposoluble	[38]
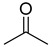	Acetone	C_3_H_6_O	Attract	Hydrosoluble/Liposoluble	[38,43]
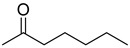	2-Heptanone	C_7_H_14_O	Attract	Liposoluble	[38,44]
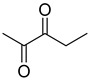	2,3-Pentanedione (low)	C_5_H_8_O_2_	Attract	Liposoluble	[45,46]
	2,3-Pentanedione (high)		Repel		[45,46]
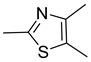	2,4,5-Trimethylthiazole (low)	C_6_H_9_NS	Attract	Liposoluble	[38]
	2,4,5-Trimethylthiazole (high)		Repel		[31,38]
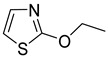	2-Ethoxythiazole	C_5_H_7_NOS	Attract	Liposoluble	[37,38]
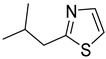	2-Isobutylthiazole	C_7_H_11_NS	Attract	Liposoluble	[37,38]
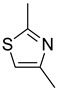	Dimethylthiazole	C_5_H_7_NS	Attract	Hydrosoluble/Liposoluble	[37,38]
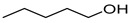	1-Pentanol	C_5_H_12_O	Attract	Liposoluble	[37,38]
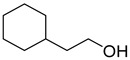	2-Cyclohexylethanol	C_8_H_16_O	Attract	Liposoluble	[37]
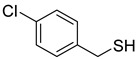	4-Chlorobenzyl Mercaptan	C_7_H_7_ClS	Attract	Liposoluble	[37]
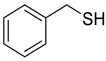	Benzyl Mercaptan	C_7_H_8_S	Attract	Liposoluble	[37]
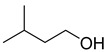	Isoamyl Alcohol (low)	C_5_H_12_O	Attract	Liposoluble	[38]
	Isoamyl Alcohol (high)		Repel		[31,41]
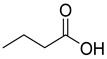	Butyric Acid	C_4_H_8_O_2_	Attract	Hydrosoluble/Liposoluble	[37]
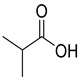	Isobutyric Acid	C_4_H_8_O_2_	Attract	Hydrosoluble/Liposoluble	[37]
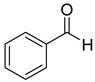	Benzaldehyde (low)	C_7_H_6_O	Attract	Liposoluble	[38,47]
	Benzaldehyde (high)		Repel		[31,38,41]
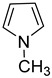	N-Methylpyrrole	C_5_H_7_N	Attract	Liposoluble	[37]
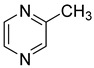	2-Methylpyrazine	C_5_H_6_N_2_	Attract	Hydrosoluble/Liposoluble	[37]
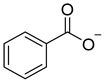	Benzoate	C_7_H_5_O_2_^−^	Attract	Hydrosoluble	[37]

## Data Availability

Not applicable.
